# Advanced vaccinomic, immunoinformatic, and molecular modeling strategies for designing Multi- epitope vaccines against the *Enterobacter cloacae complex*

**DOI:** 10.3389/fimmu.2024.1454394

**Published:** 2024-08-16

**Authors:** Hassan H. Alhassan

**Affiliations:** Department of Clinical Laboratory Sciences, College of Applied Medical Sciences, Jouf University, Sakaka, Saudi Arabia

**Keywords:** *Enterobacter cloacae complex*, vaccine construct, vaccinomics, molecular dynamics simulation, immunoinformatic

## Abstract

The increasing and ongoing issue of antibiotic resistance in bacteria is of huge concern globally, mainly to healthcare facilities. It is now crucial to develop a vaccine for therapeutic and preventive purposes against the bacterial species causing hospital-based infections. Among the many antibiotic- resistant bacterial pathogens, the *Enterobacter cloacae complex* (ECC) including six species, *E. Colcae*, *E. absuriae*, *E. kobie, E. hormaechei, E. ludwigii*, and *E. nimipressuralis*, are dangerous to public health and may worsen the situation. Vaccination plays a vital role in the prevention of infections and infectious diseases. This research highlighted the construction and design of a multi-epitope vaccine for the *E. cloacae* complex by retrieving their complete sequenced proteome. The retrieved proteome was assessed to opt for potential vaccine candidates using immunoinformatic tools. Both B and T-cell epitopes were predicted in order to create both humoral and cellular immunity and further scrutinized for antigenicity, allergenicity, water solubility, and toxicity analysis. The final potential epitopes were subjected to population coverage analysis. Major histocompatibility complex (MHC) class combined, and MHC Class I and II world population coverage was obtained as 99.74%, and 98.55% respectively while a combined 81.81% was covered. A multi-epitope peptide-based vaccine construct consisting of the adjuvant, epitopes, and linkers was subjected to the ProtParam tool to calculate its physiochemical properties. The total amino acids were 236, the molecular weight was 27.64kd, and the vaccine construct was stable with an instability index of 27.01. The Grand Average of Hydropathy (GRAVY) (hydrophilicity) value obtained was -0.659, being more negative and depicting the hydrophilic character. It was non-allergen antigenic with an antigenicity of 0.8913. The vaccine construct was further validated for binding efficacy with immune cell receptors MHC-I, MHC-II, and Toll-like receptor (TLR)-4. The molecular docking results depict that the designed vaccine has good binding potency with immune receptors crucial for antigen presentation and processing. Among the Vaccine-MHC-I, Vaccine-MHC-II, and Vaccine-TLR-4 complexes, the best-docked poses were identified based on their lowest binding energy scores of -886.8, -995.6, and -883.6, respectively. Overall, we observed that the designed vaccine construct can evoke a proper immune response and the construct could help experimental researchers in the formulation of a vaccine against the targeted pathogens.

## 1 Introduction

The *Enterobacter cloacae complex* (ECC) consists of a group of species including *E. Colcae*, *E. absuriae*, *E. kobie, E. hormaechei, E. ludwigii*, and *E. nimipressuralis*. The cluster-based composition of the ECC includes 13 clusters (C-I TO C-XIII). Clusters C-III, VI, and VIII are known to be isolated from human samples (1). Being a member of the family *Enterobacteriaceae*, it is a facultative anaerobe, gram-negative rod-shaped bacteria (2). The *E. Cloacae* complex is known to be a nosocomial pathogen leading to various infections such as lower respiratory infection, septicemia, urinary tract, and pneumonia (3). The incident rate of the ECC is estimated to range from 65% to 75%. Being the third main drug-resistant species with a role in nosocomial infection, the ECC can develop many genetic variations related to drug resistance genes and is known to attain multidrug resistance (4). The AmpC gene of the ECC determines the production of B-lactamase, which is known to exhibit resistance against amoxicillin, ampicillin, cephalosporin, and cefoxitin (known as antibiotics) (5). The reason behind the ECC being multidrug-resistant is the extensive use of antibiotics (6). Among the ECC species, *E. hormaechei*, being a causative agent, is more likely to cause infections (7). According to a report in which 36 ECC strains were isolated, 94.44% were *E. hormaechei* species while only 2.77% of the species were observed to be *E. Kobei* (8). These pathogens are saprophytic and have shown their presence in sewage, soil (5), and the human gastrointestinal tract, their most common reservoir (9). Outbreaks of *E. cloacae* infection were reported in neonatal units. Analysis of 26 reported studies revealed that 16 were bloodstream infections (BSI) and 2 of them were due to the medication (10). In 1998, during an outbreak in South Africa, 9 deaths were reported. Later, a few outbreaks were also reported with a mortality rate of 39% (11) and 5-10% of the infections were reported as ICU (intensive care unit) infections (12). Reported data of nosocomial infections suggest that 5% of the total cases were septicemia, 4% were urinary tract infections, 5% were pneumonia, and 10% were postsurgical peritonitis due to *E. cloacae (* 13).

As far as the genome of the ECC is concerned, *E. absuriae* is comprised of a 4.81 single chromosomal DNA that is circular in shape. GC content for *E. absuriae* is reported to be 55.47% (14). Only a single chromosomal DNA along with the plasmids makes up the genome of *E. kobeii* with a total of 4.75Mbp. The 4.89 Mbp genome sequence of *E. hormaechei* contains only 1 plasmid along with 3 scaffolds*. E. ludwigii*, like *E. kobeii*, contains a plasmid and a single chromosomal DNA as its genome of 4.95 Mbp. The *E. nimipressuralis* genome is 4.98 Mbp as observed, based on 18 scaffolds of DNA that encode for 4875 genes. Observed GC content for *E. kobie, E. hormaechei, E. ludwigii*, and *E. nimipressuralis* is 55.43%, 55.1%, 55.43, and 55.1% respectively (15). The ECC, being a hospital-acquired infection, is also known for its multi-drug resistance, so there is a need to tackle this problem. Immunoinformatic and computational techniques are being used to design vaccines and they promise efficacy at a very low cost within a short period (16). Computational techniques involve reverse vaccinology and core proteome analysis for designing vaccines against the multidrug-resistant ECC which provokes nosocomial infections. Multi-epitope-designed vaccines are considered more effective with no side effects in contrast to conventionally manufactured vaccines (16). Hence this research study is a depiction of a multi-epitope peptide vaccine against the ECC using computational approaches. The extracted proteins were analyzed to ascertain potential candidates for epitope mapping and were further prioritized to focus on those with a tendency to be a part of a vaccine construct that led towards molecular docking to assess the interaction between the vaccine and molecules/receptors of the human body. Furthermore, the process included calculating the molecular dynamic simulation and estimating the binding free energy.

## 2 Research methodology

### 2.1 Pan-genome analysis for ECC

**Figure 1:**
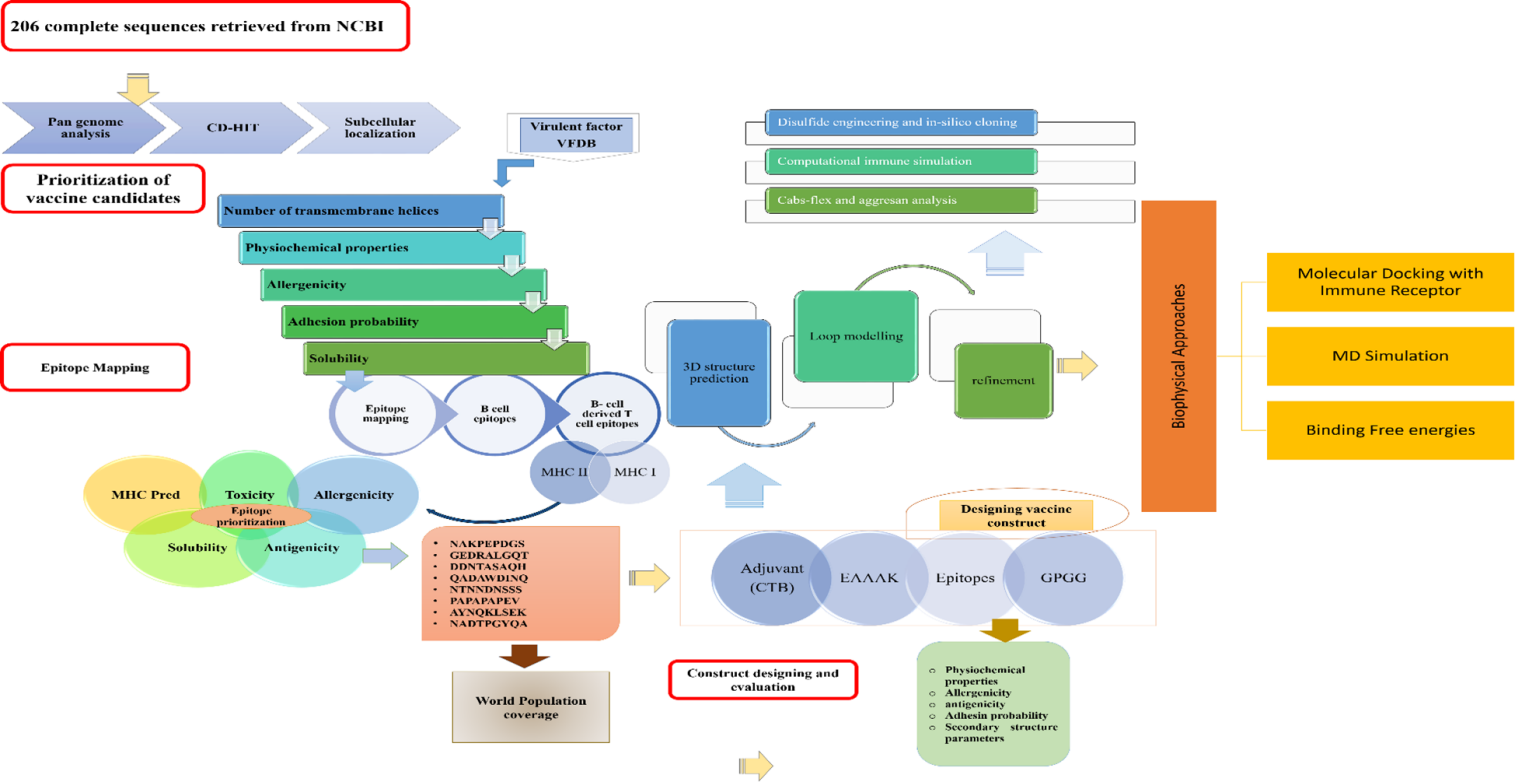
Designed methodology to be followed for multi-epitope vaccine designing.

Complete methodology flow and process used to design Multi epitope vaccine against ECC is shown in **Figure 1**. Pan-genome analysis was conducted by retrieving the complete coding genome sequences for all 6 species of ECC from the National Centre for Biotechnology Information (NCBI) (17). BPGA software (18) was used to get the core proteome from the retrieved sequences and these were subjected to CD-HIT analysis, selecting a 90% threshold and removing repetitive protein sequences which resulted in non-redundant sequences. CD-HIT (cluster database) was used for the analysis of the core proteomes and the sequences observed as having a higher identity were removed. Subcellular localization of the protein sequences was done to predict the function and location of the respective protein. Subcellular localization of the ECC non-redundant protein sequences was performed by Bacterial Protein Subcellular localization prediction tool (PSORT-B) (19). The outer membrane, periplasmic, and extracellular sequences were separated and subjected to various analyses, eventually used for epitope mapping. The initial step performed for protein shortlisting was the evaluation of transmembrane helices lying in the protein by TMHMM 2.0 (an online tool to predict transmembrane helices). The physiochemical properties including molecular weight, instability and therapeutical index, and GRAVY value (score for hydrophilicity) were predicted using the ProtParam tool in Expasy. A molecular weight less than 100kd, an instability index below 45, and a more negative GRAVY score were included in the selection criteria, leading to an allergenicity check using Allertop 2.0 (20) and an antigenicity check using Vaxijen (21) with a threshold of 0.5. Adhesion probability using Vaxign was also calculated for the protein sequences. Those predicted as allergens and non-antigens were discarded. Protein Basic Local Alignment search tool (BLASTp) against the *Lactobacillus* species and humans was also performed.

### 2.2 Epitope mapping

Using the shortlisted proteins, epitope prediction was done using the Immune Epitope Database (IEDB) (22). The predicted peptides with the potential to be epitopes were T cell epitopes derived from B cell epitopes. The shortlisted proteins were used as input sequences in the IEDB’s linear B cell epitope prediction to predict B cell epitopes. The B cell epitope mapping was followed by major histocompatibility complex (MHC) II binding T cell epitopes and then MHC I binding T cell epitopes. A complete alleles reference set was used to predict both MHC I and II binding T cell epitopes. The binding affinities of the predicted peptide sequences behaving as B cell derived T cell epitopes were further analyzed using MHC-Pred. Epitopes with an IC50 value less than 100 were regarded as good binders and subjected to further scrutiny, including antigenicity with a threshold of 0.7 (epitopes with a higher score are considered more antigenic with a higher probability to bind with MHCs), allergenicity (the check that depicts the probability of an epitope to be non-allergen or allergen), solubility (epitopes with low solubility are considered unstable while those with good water solubility are potentially good stable epitopes for vaccine development), and toxicity. Only epitopes that were antigens and non-allergens, had good solubility in water, and were non-toxins were chosen to map a peptide-based multi-epitope vaccine construct.

### 2.3 Population coverage

The shortlisted epitopes for the population were evaluated against the set of different alleles covering all the geographic regions of the world using the IEDB’s population coverage. Combined global population coverage and population coverage for both MHC I and II were calculated while the region-wise population coverage was also calculated.

### 2.4 Multi-epitopes peptide-based vaccine designing

The epitopes that were shortlisted were then used to map a vaccine construct. GPGPG linkers were utilized to connect an epitope to an epitope and the EAAAK linker was employed to link epitopes to an adjuvant. Cholera toxin B (CTB) was used as an adjuvant. The ProtParam tool in Expasy was used to map the physiochemical properties of the vaccine construct using epitopes (23). The properties evaluated were the molecular weight, GRAVY, instability, and aliphatic index. The vaccine construct was then modeled into its 3D structure via Scratch Protein Predictor (24). The 3D structure obtained was subjected to loop modeling using Galaxy Loop and refined using Galaxy Refine (25).

### 2.5 Disulfide engineering

The process of inserting disulfide bridges/bonds in s vaccine model to make the structure more stable is referred to as disulfide engineering. Regions possessing instability are first identified and then mutated into cysteine residues which requires the disulfide bond insertion. This step was performed in the online server Disulfide By Design (26), resulting in the mutant model.

### 2.6 Aggrescan and CABS-flex analysis

Attaining structural stability is critical in vaccine design. Aggregation-prone regions were investigated using Aggrescan3D (27) and then the structure was subjected to CABS-flex 2.0. The number of cycles selected was 50 and 8335 RNG seed. The selected temperature range was 1.40, while the restraints for the global side chain and global C-alpha were 1.0 (28). CABS-flex is a coarse grind simulation used to infer and model the flexibility of the structure.

### 2.7 *In silico* cloning

The vaccine model was reverse-translated into its DNA sequence following the phenomenon of codon optimization in JCat (Java codon adaptation tool) (29). The GC content and CAI value were obtained and used to evaluate the sequences against the *E. coli* k12. The reversely translated vaccine construct (DNA sequence) was then cloned in a pET-28a (+) expression vector and this cloning was performed using the SnapGene tool.

### 2.8 Computational immune simulation

The multi-epitope vaccine model was analyzed for its immunogenicity and capability of inducing immune responses in the human body on a C-ImmSim server (30). This server is used to predict the immune-epitope interaction based on machine learning techniques.

### 2.9 Molecular docking

Molecular docking tends to predict the binding interaction and affinities of a vaccine model with body receptors in humans. In this study, three receptors, MHCI, MHCII, and TLR-4 (toll-like receptor-4) were used for docking. The multi-epitope vaccine model was docked one by one with these three receptors using the online tool ClusPro 2.0 (31) based on the Fast Fourier Transform (FFT) method.

### 2.10 Molecular dynamic simulations

The complexes resulting from molecular docking were analyzed for molecular simulations using Amber18 software. The production run selected was 50ns to carry out the procedure in an aqueous solution. The complexes were incorporated in a Transferable Intermolecular Potential with 3 points (TIP3P) water box and the force field for it was Force Field used with TIP3P model (FF14SB) and the padding distance maintained was 12 Å. The system was set to be neutralized using Na+ ions. The concentration of the atoms added to the system was reduced: hydrogen atoms to 500 steps, carbon alpha atoms to 1000 steps, non-heavy atoms to 300 steps, and the solvation box to 1000 steps. The temperature of the system was maintained by system heating to 300k for 20ps. To stabilize the system, it was slowed down to 100ps. Constant-pressure, constant-temperature ensemble involved pressure maintenance for 50ps, leading to the completion of the production run of 50ns for 2fs. Trajectory analyses were obtained using Amber CPPTRAJ. Molecular Mechanics with Poisson Boltzmann or Generalized Born and Surface area (MMPB/GBSA) were calculated for the docked complex by the MMPBSA.py package in the Amber18 program and the binding free energies between the molecules were calculated.

## 3 Results

### 3.1 Pan-genome analysis for ECC

Pan-genome analysis was applied to 206 complete sequences of the *E. cloaca* complex retrieved from NCBI, resulting in 21936 core proteome sequences. BPGA, a genome analysis tool, was used for the retrieval of the core proteomes. CD-HIT analysis with a threshold of 90% was carried out to eradicate duplicate/repetitive sequences and resulted in 1978 non-redundant sequences. The unique non-redundant sequences were subjected to PsortB for subcellular localization (predicting the protein location). The outer membrane, periplasmic, and extracellular protein sequences were selected to proceed with the design of the multi-epitope vaccine, which were 46, 67, and 11 in number respectively.

All these protein sequences were further analyzed for bacterial virulence factor using Virulence Factor Database (VFDB) resulting in unique sequences with more than 35 identities and bit scores above 100. Of the total protein sequences, 27 were shortlisted with 13 outer membrane sequences, 9 periplasmic sequences, and 5 extracellular protein sequences. The shortlisted protein sequences were inspected for the existence of transmembrane helices by TMHMM. The sequences lacking transmembrane helices or having only one underwent further analysis. Only one (an outer membrane sequence) of the 27 protein sequences was discarded because its number of transmembrane helices was 3. To select the protein marking the criteria of potential candidates for a vaccine, the physicochemical properties of the remaining 26 protein sequences were analyzed. The ProtParam tool in Expasy was used to calculate molecular weight (should be less than 100kd), theoretical index (if less than 7 indicates the acidic nature of the protein), GRAVY (hydrophilicity, should be more negative to indicate the hydrophilic character of the protein), and instability index (an instability index less than 45 indicates the stability). Five proteins were marked unstable with an instability index of 46.43, 46.53, 40.28, 43.43, and 50.28, respectively, and were discarded. The remaining 21 protein sequences were then checked for their antigenicity using Vaxijen. The threshold selected was 0.5. Nine protein sequences with an antigenicity score of less than 0.5 were considered non-antigen and were discarded, leaving the 12 protein sequences that were subjected to the AllerTop tool for allergenicity check, of which three were predicted as allergens. The non-allergenic protein sequences were analyzed for the adhesion probability using Vaxign with a threshold of 0.5. All the proteins had adhesive properties (**Table 1**). Finally, the solubility check was applied to the remaining nine protein sequences using the Innovagen tool, of which four of them had good water solubility (**Table 1**). Human BLASTp and lactobacillus species BLASTp were run against these shortlisted proteins, but no significant similarity was found. When BLASTp was run against the protein sequences core/6370/1/Org1_Gene1656, core/828/2/Org2_Gene1097, core/5290/2/Org2_Gene1489, and core/14118/9/Org9_Gene2539, they showed 100% similarity for flagellar hook-associated protein FlgL, TonB-dependent siderophore receptor, porin OmpA, and flagellar basal body rod protein FlgB, respectively.

**Table 1:** Protein sequences with their physiochemical properties.

IDs	LENGTH	identity	bit score	TMHMM	M.W	STABILITY INDEX		GRAVY	THEORETICAL INDEX	ANTIGENICITY	Allergenicity	adhesion probability	solubility
core/6370/1/Org1_Gene1656	317	53	852	0	34.3	28.27	stable	-0.457	4.61	0.5012	non-allergen	0.631	soluble
core/828/2/Org2_Gene1097	749	78	3071	0	83	31.88	stable	-0.636	5.55	0.7625	non-allergen	0.803	soluble
core/5290/2/Org2_Gene1489	351	80	1389	0	37.6	26.28	stable	-0.376	5.32	0.7176	non-allergen	0.513	soluble
core/14118/9/Org9_Gene2539	139	81	573	0	15.2	27.27	stable	-0.423	5.35	0.6599	non-allergen	0.566	soluble

### 3.2 Epitope mapping and prioritization

In this step, by using the shortlisted protein sequences T cell epitope (B cell-derived) prediction was performed using the IEDB. The IEDB’s B cell epitope prediction was used for B cell epitope mapping and 29 peptides were predicted, behaving as B cell epitopes with a threshold of 0.5. The predicted peptides behaving as B cell epitopes were the input sequences for the prediction of T cell epitopes. MHC-II binding T cell epitopes were predicted first for each predicted B cell epitope, followed by the process of predicting MHC-I binding T cell epitopes from the predicted MHCII binding epitopes. The obtained 35 T cell epitopes (B cell-derived) were subjected to various analyses to shortlist potential epitopes to design a vaccine construct. MHC-pred was applied to these epitopes to predict their binding affinity, the DRB*0101 allele was selected, and epitopes were picked on the basis of their IC50 value. A calculated IC50 value of less than 100 was acceptable, depicting the epitopes as good binders. Antigenicity (threshold of 0.7) and allergenicity checks were then applied to the epitopes with an IC50 value less than 100. Of these, 15 epitopes were non-antigen with a threshold value less than 0.7, and the 10 that were predicted as allergens were discarded. The remaining 11 epitopes, being antigenic and non-allergen, were further subjected to Innovagen and ToxinPred for their solubility and toxicity check. Two epitopes showed poor water solubility and one was predicted to be a toxin. The remaining eight epitopes met the criteria of potential epitopes to be used as part of a multi-epitope peptide vaccine. The details of the shortlisted epitopes are shown in **Table 2**.

**Table 2:** Shortlisted epitopes and their properties.

Protein ids	B cell epitopes	MHC II binding	P.R	MHC I binding	P.R	MHC pred	IC50 value	Antigenicity		Allergenicity	solubility	toxicity
core/6370/1/Org1_Gene1656	TSNAKPEPDGSAPET	TSNAKPEPDGS	40	NAKPEPDGS	15	NAKPEPDGS	10.57	1.1774	antigen	non-allergen	soluble	non-toxin
core/6370/1/Org1_Gene1656	SLGEDRALGQTQQ	SLGEDRALGQT	13	GEDRALGQT	2.3	GEDRALGQT	52.97	1.0867	antigen	non-allergen	soluble	non-toxin
core/828/2/Org2_Gene1097	LADDNTASAQHE					DDNTASAQH	32.43	1.5066	antigen	non-allergen	soluble	non-toxin
core/828/2/Org2_Gene1097	DKTQADAWDINQGHQSDRTGAYADTLPAGREGVENKDI	TQADAWDINQG	4.1	QADAWDINQ	4.5	QADAWDINQ	10.09	0.8036	antigen	non-allergen	soluble	non-toxin
core/828/2/Org2_Gene1097	NLYAGDTQNTNNDNSSSGLV	QNTNNDNSSSG	36	NTNNDNSSS	9.9	NTNNDNSSS	75.68	1.8953	antigen	non-allergen	soluble	non-toxin
core/5290/2/Org2_Gene1489	FGQQEEAAPVVAPAPAPAPEVQT	VAPAPAPAPEV	3.6	APAPAPAPEV	0.12	PAPAPAPEV	7.89	1.121	antigen	non-allergen	soluble	non-toxin
core/5290/2/Org2_Gene1489	RIGSDAYNQKLSEKRAQ	AYNQKLSEKRA	4.8	AYNQKLSEK	0.14	AYNQKLSEK	35.65	1.6336	antigen	non-allergen	soluble	non-toxin
core/14118/9/Org9_Gene2539	ANADTPGYQARDID	ANADTPGYQAR	38	NADTPGYQAR	0.48	NADTPGYQA	14.55	1.5818	antigen	non-allergen	soluble	non-toxin

### 3.3 Population coverage

**Figure 2:**
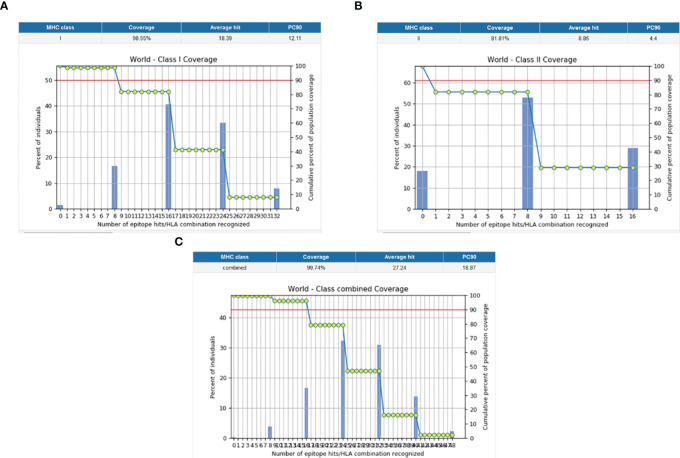
Graphs depicting the population coverage of each epitope against a set of alleles all over the world. The cumulative percentage of population coverage of epitopes is given, and the bars depict the population coverage of each epitope. **(A)** MHC-I world population coverage. **(B)** MHC-II world population coverage. **(C)** Class combined population coverage.

**Figure 3:**
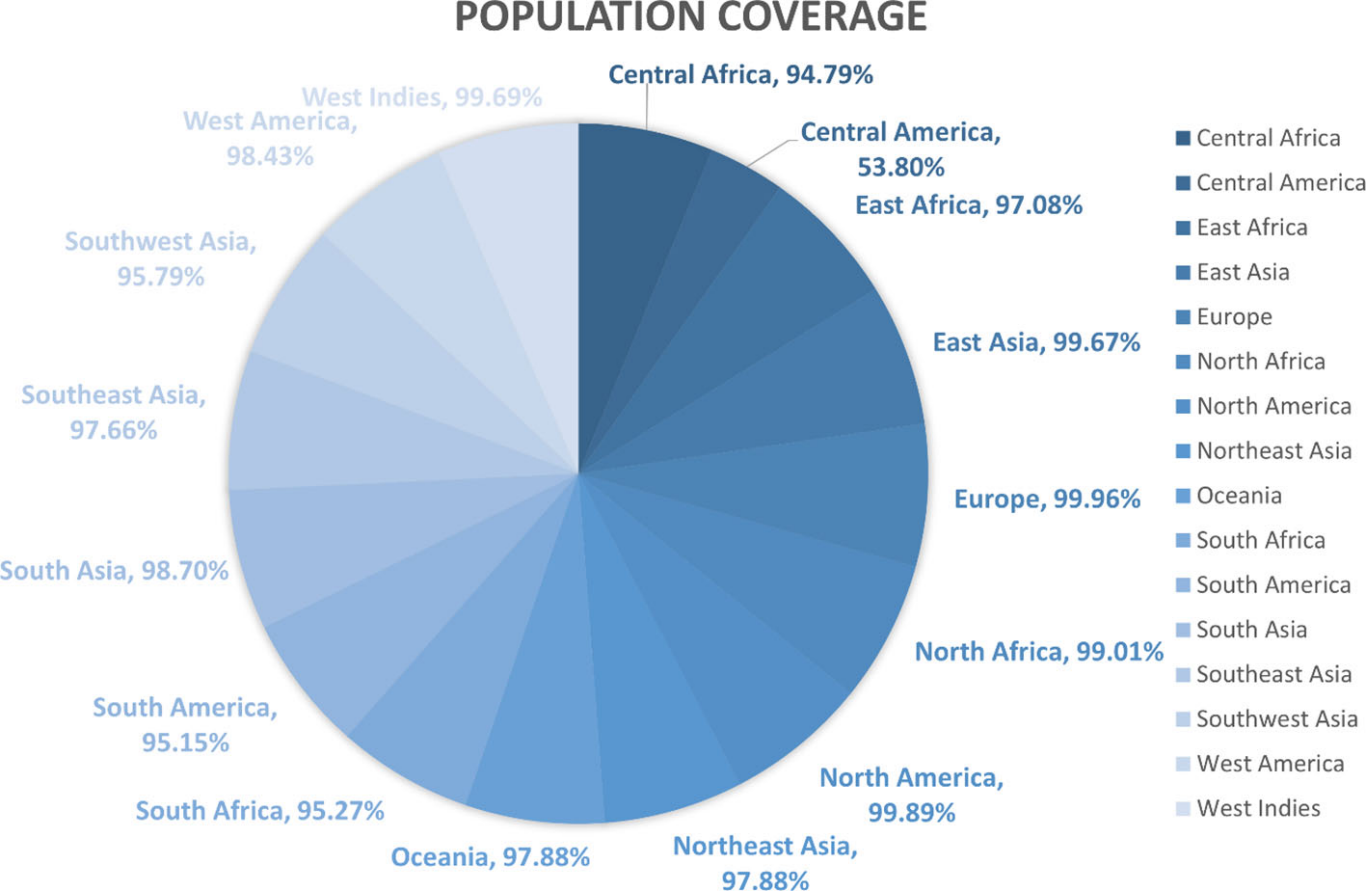
Region-wise class combined population coverage obtained from the IEDB. The estimated class combined world population coverage from regions all over the world is given. The highest world population coverage recorded was 99.96% in Europe while the lowest recorded was 53.80% in Central America.

The population coverage of the final potential epitopes was analyzed using population coverage in the IEDB to estimate their overall population coverage for the complete set of alleles. The MHC class combined world population coverage obtained was 99.74% (**Figure 2C**), for MHC I it was 98.55% (**Figure 2A**), and for MHC II it was 81.81% (**Figure 2B**). The class combined population coverage based on geographic regions of the world was also obtained (**Figure 3**).

### 3.4 Vaccine design

**Figure 4:**
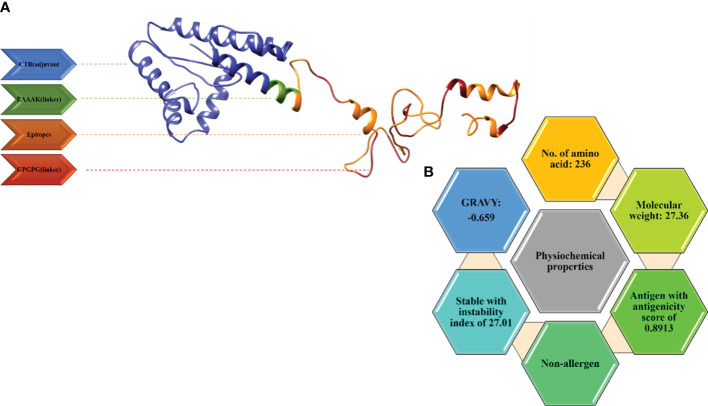
**(A)** 3D model for the vaccine along with its components. **(B)** Physiochemical properties of the multi-epitope vaccine.

The idea of a multi-epitope peptide vaccine is based on the presence of linkers joining epitope to epitope and epitope to adjuvant. The adjuvant is a peptide sequence used to boost the working capability of the vaccine. In this vaccine, we linked our shortlisted potential epitopes using a GPGPG linker. The adjuvant, Cholera toxin B (MIKLKFGVFFTVLLSSAYAHGTPQNITDLCAEYHNTQIYTLNDKIFSYTESLAGKREMAIITFKNGAIFQVEVPGSQHIDSQKKAIERMKDTLRIAYLTEAKVEKLCVWNNKTPHAIAAISMAN), was linked by an EAAAK linker to the epitopes. The vaccine construct consisting of the adjuvant, epitopes, and linkers was subjected to the ProtParam tool by Expasy to calculate its physiological properties. The total amino acids were 236 and the molecular weight was 27.64kd, which is considered good. The vaccine construct was stable also with an instability index of 27.01. The GRAVY (hydrophilicity) value obtained was -0.659 and, being more negative, depicts the hydrophilic character. The vaccine construct was non-allergenic and antigenic with an antigenicity as shown in **Figure 4B**.

### 3.5 Structure modeling

**Figure 5:**
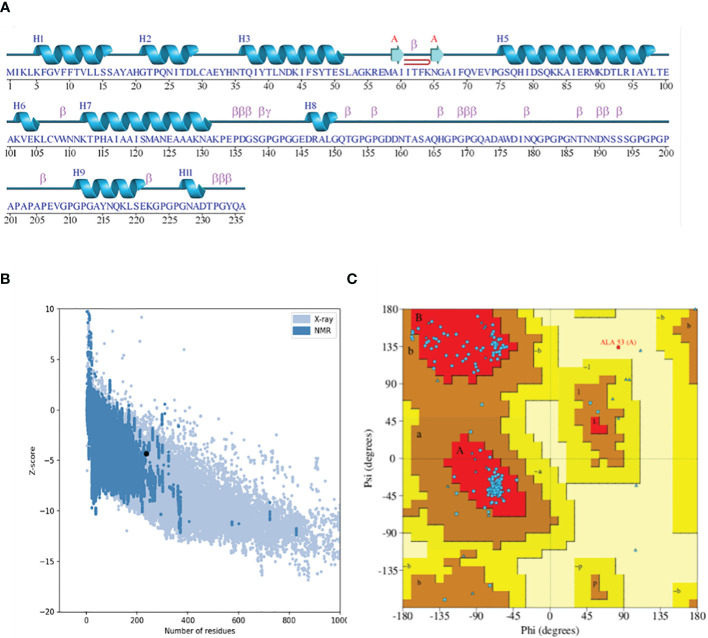
**(A)** Secondary structure highlighting the strands and helices. **(B)** Plot depicting the Z-score plot (a standard score giving the probability of a score within the normal distribution). **(C)** Ramachandran plot showing that 90% of the residues were in the most favored (residues in red) regions.

The 3D structure modeling of the vaccine construct was the next step, leading to the analysis of loops and the structure. The Scratch Protein Predictor was employed for the prediction of 3D model of the vaccine. The 3D model was then subjected to loop modeling. We observed a total of eight loops in the model and submitted the model to Galaxy Loops in the Galaxy web server loop modeling to predict the residues in the loop regions. This step was followed by the refinement of the vaccine model, resulting in five models, The first model was predicted and refined by Galaxy Refine in Galaxy Web with a GDT-HA value of 0.9460 and a root mean square deviation (RMSD) value of 0.422. The 3D model for the multi-epitope vaccine is shown in **Figure 4A**. The secondary structure (**Figure 5A**) for the vaccine model and Ramachandran plot was also predicted and analyzed using PDBsum Generate. The secondary structure of the vaccine model consisted of two strands, one beta-hairpin, and one sheet with 11 helices, seven helix-helix interactions, one gamma, and 22 beta turns. The Ramachandran plot (**Figure 5C**) shows that out of non-glycine and non-proline residues 168 residues are present in most favored regions. Additionally, the allowed regions contained 11 residues while the disallowed regions contained only one. Furthermore, glycine and proline residues were 30 and 24 in number respectively. The evaluated Z-score was -4.31, indicating the quality of the vaccine model (**Figure 5B**).

### 3.6 Disulphide engineering

**Figure 6:**
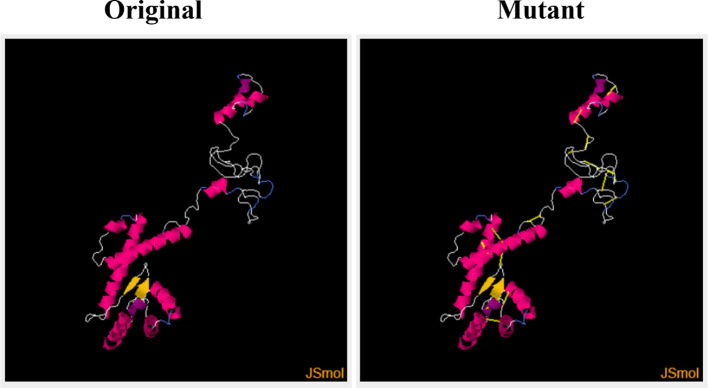
Original (wild) and the mutant (generated by Disulphide By Design by incorporating disulphide bonds to mutate into cysteine residues) vaccine models.

This step consists of mutating the pairs of amino acid residues into cysteine residues through the insertion of disulphide bonds into the vaccine model, resulting in the formation of a mutant model (**Figure 6**). The analysis of the stability of the vaccine model and mutating it to introduce stability at the regions where instability had been observed was the need for disulphide engineering. A total of 17 residue pairs were observed to be unstable regions and were mutated into cysteine residue as shown in **Table 3**.

**Table 3:** Residue pairs along with the X3 and CAI value obtained by Disulfide By Design.

Residue 1		Residue 2		X3	Kcal/mol
4	LEU	7	GLY	110.12	3.14
7	GLY	32	GLU	-72.61	5.93
22	THR	39	TYR	-101.95	3.67
34	HIS	38	ILE	-116.41	4.49
62	THR	121	SER	-74.7	5.11
92	THR	118	ALA	70.41	5.42
96	ALA	122	MET	-76.4	3.29
138	SER	141	GLY	123.73	4.18
163	SER	168	PRO	-92.74	1.39
166	HIS	195	GLY	-105.86	4.8
171	GLY	194	SER	-95.53	3.6
173	ALA	196	PRO	-96.9	5.81
182	PRO	203	ALA	-70.62	4.77
184	PRO	197	GLY	-84.8	2.86
205	ALA	208	VAL	105.77	5.02
210	PRO	213	GLY	110.78	3.15
226	PRO	229	ALA	121.56	3.85

### 3.7 Aggrescan and CABS-flex analysis

**Figure 7:**
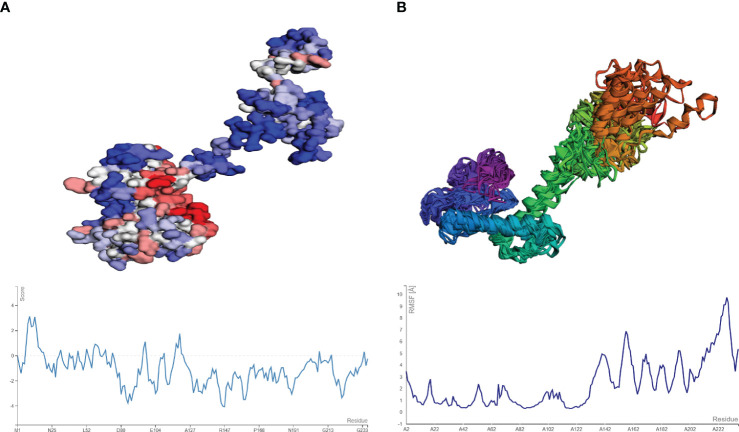
**(A)** Model and plot differentiating the aggregation-prone (having a score above zero) regions in the vaccine model. **(B)** resulting model from CABS-flex and the fluctuation plot (highest RMSF score of 9.2 Å).

The aggregation-prone residue was analyzed using Aggrescan followed by the coarse grind simulation for the flexibility of the vaccine modes using CABS-flex. The aggregation-prone regions observed had a score of more than 0 (positive value) with those with negative values considered soluble residues, as shown in **Figure 7A**. The average score obtained from Aggrescan analysis was -0.954, indicating the residues with normalized solubility. The CABS-flex analysis was performed on the structure (pdb) obtained from the Aggrescan analysis, resulting in the prediction of 10 models. The fluctuation plot in **Figure 7B** represents the RMSF value for the residues. The highest RMSF calculated was 9.74 Å at residue 228.

### 3.8 *In silico* clonin*g*

**Figure 8:**
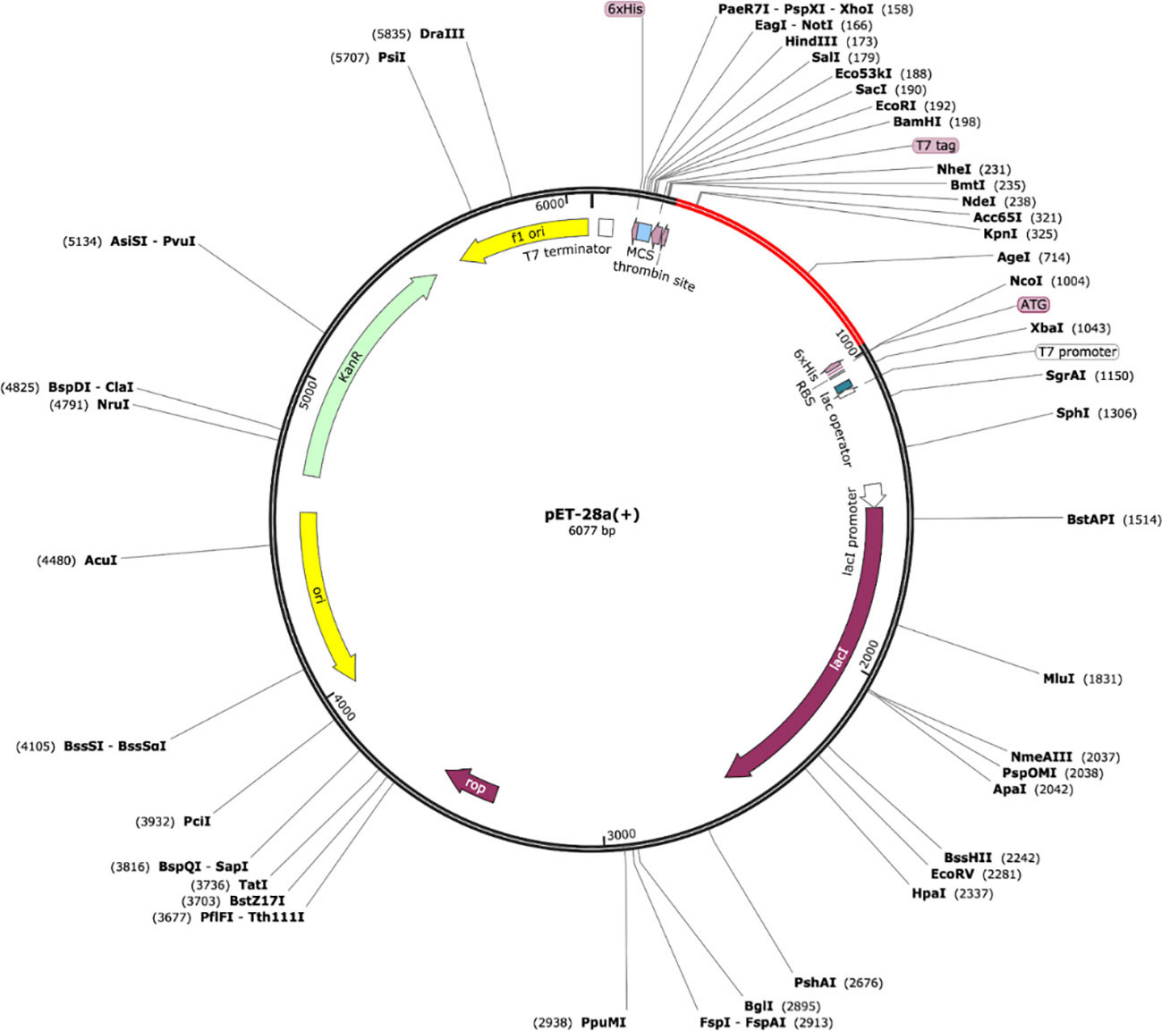
Vaccine model (shown in red) expressed in the expression system [pET-28a (+)].

*In-silico* cloning refers to the phenomenon of expressing the vaccine sequence in an expression system e.g., *E. coli* K12. For this, peptide-based sequences of the vaccine were subjected to a reverse translation using the JCat tool, resulting in an improved sequence (DNA) through codon optimization with a GC content of 54.80 and CAI value equal to 1.0. The DNA sequence of the peptide vaccine was then expressed in expression vector pET-28a (+) using SnapGene as depicted in **Figure 8**.

### 3.9 Computational immune simulations

**Figure 9:**
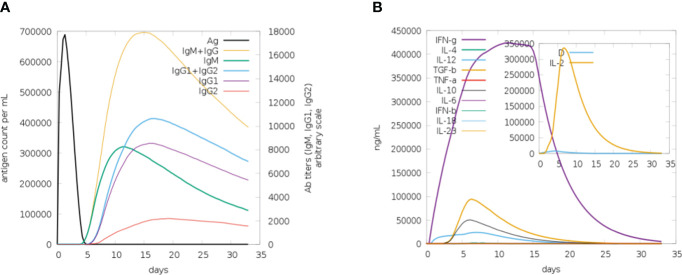
**(A)** Plot for the rate of production of immunoglobulins in response to the vaccine, upon the first week of induction the rate of production remains high. **(B)** Plot depicting the rate of interleukins being induced in response to vaccine. The increase in the production of IFN-g in the first 2 weeks can be seen (purple).

Computational immune simulations were performed on the C-ImmSim server to observe immune responses induced by the multi-epitope vaccine. The vaccine was effective enough to induce immunity which was progressed by the induction of several immunoglobulin and interleukin productions. **Figure 9A** highlights the immunoglobulin production against the vaccine. There was an increase over a number of days, resulting in higher production, especially of IgM+IgG. After 15-20 days, a decrease in the production of immunoglobulins can be seen. Similarly, there was an increase in the production of interleukins and IFN-g from day 1 and it increased in the first week, followed by decreased production, and then the body stopped producing interleukins after 20 days. A higher rate of IFN-g induction was observed and its production started decreasing after 20 days (**Figure 9B**).

### 3.10 Molecular docking

**Figure 10:**
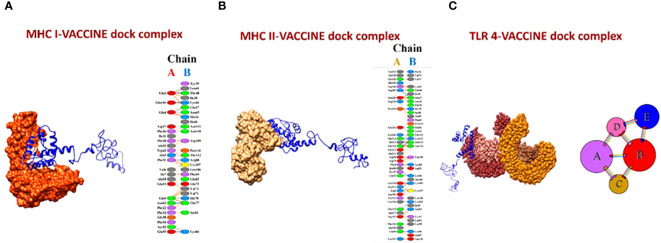
**(A)** MHC I-vaccine and the interaction in chain A (receptor) and chain B (vaccine) of the complex. **(B)** MHC II-complex and the interaction between chain A (receptor) and chain B (vaccine) of the complex. **(C)** TLR-4-complex and its five interacting chains.

The molecular docking of the vaccine model was carried out using three receptors, namely MHCI, MHCII, and TLR-4, by ClusPro 2.0. The MHCI-vaccine, MHCII-vaccine, and TLR-4-vaccine interactions were predicted and analyzed. In total, 10 models for each dock complex were obtained along with their balanced calculated energies. Dock complexes for the three receptors are shown in **Figure 10**. The interaction between the residues of the receptor (chain A) and vaccine (chain B) for both MHC I and II docked complexes is shown in **Figures 10A, B**. The TLR-4-complex is comprised of five chains (**Figure 10C**) and the interaction between them is given in **Supplementary Table S1**. The energies calculated for the MHCI dock complex, MHCII dock complex, and TLR-4 dock complex are listed in **Supplementary Tables S2**-**S4** respectively. Models of dock complexes were arranged on the basis of cluster size, while energies calculated and listed are of the model present at the center of the cluster and the neighboring member with the lowest energy within the cluster. The score at the center for the best model chosen was -849.8 and the lowest energy for the MHC I-vaccine complex was -886.7. For MHC II, the score at the center and the lowest energy were estimated as -900.1 and -995.1 respectively. The best selected TLR 4-vaccine complex showed a score at the center of -798.5 and the lowest energy of -883.6.

### 3.11 Molecular dynamic simulation

**Figure 11:**
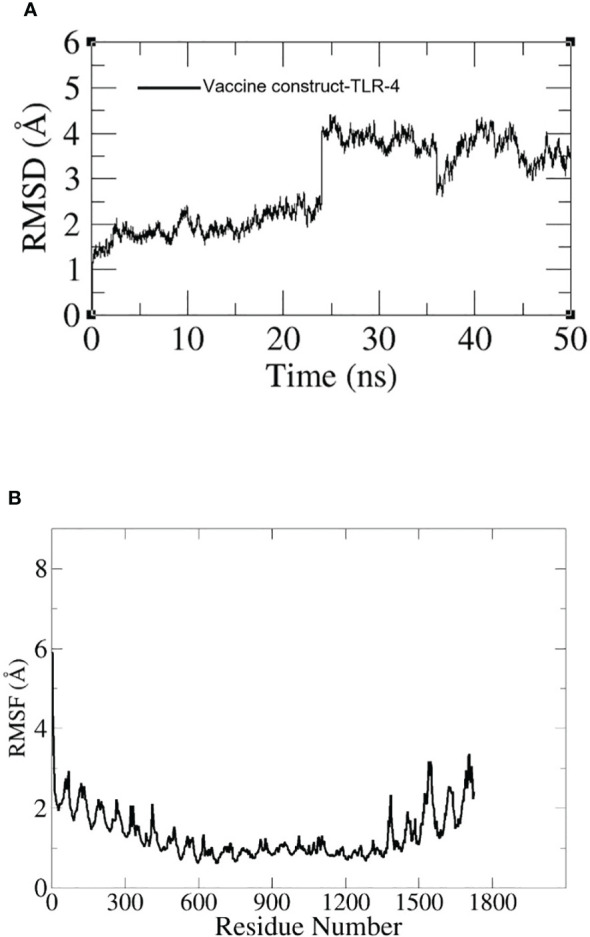
**(A)** RMSD graph for TLR-4-construct. **(B)** RMSF graph for TLR-4-construct.

To assess the dynamics of the docked complexes, molecular dynamic simulation was done. The RMSD and RMSF of carbon alpha atoms for the total amino acid residues of the proteins were analyzed for 100 nanoseconds. In the case of the TLR-4 vaccine construct, the RMSD graph showed a first deviation of 1.1 (Å) and then increased to 4.5 (Å) at 25 ns. Within 20 to 30 ns, the fluctuation increased rapidly but by the end of simulation, it showed stability as shown in **Figure 11A**. Additionally, the RMSF of the docked complexes was assessed for the estimation of the binding stability. In the RMSF plot we observed little fluctuation which might have reflected the continuous interaction between the vaccine and immune cell receptors. The RMSF graph is shown in **Figure 11B**.

### 3.12 Estimation of binding free energies

To validate the docking results binding free energies were estimated. In MM-GBSA analysis, different energy parameters such as Van der Waals force (VDWAALS) and binding free energies were checked. In the case of human TLR-4 vaccine molecules, -23.9861 kilocalories per mole delta total net binding free energy was calculated. A negative energy value represents the best binding ability of vaccine molecules with the immune cell receptors (TLR-4). The complex with greater negative binding free energy is considered to be more stable. Stronger binding energies depict the stronger forces of attraction between the receptor and the vaccine, aiding in stabilizing the complex (32). The different energy parameters and their calculated energy values are tabulated in **Table 4**.

**Table 4:** Binding free energies estimation.

Energy Parameter	TLR-4-Vaccine Complex
MM-GBSA	
VDWAALS	-33.5184
EEL	-153.63
EGB	167.4479
ESURF	-4.2856
Delta G gas	-187.149
Delta G solv	163.1624
Delta Total	-23.9861

## 4 Discussion

The ECC, consisting of 6 species, is known to be a causative agent, causing nosocomial infection associated with the bloodstream. The ECC has attained the ability of producing AmpC β-lactamase which is associated with antibiotic resistance (5). To control this infection, vaccines are a better solution. The conventional vaccine design methods are less proficient than novel computational and immunoinformatic technique-based vaccine design in terms of time cost and effectiveness. Designing a vaccine based on epitopes using immunoinformatics is considered safer, better, and more stable (16). The use of imunoinformatics in the area of cancer is also a proficient method to design a multi-epitope vaccine to activate humoral immune response (33). In our study, 206 coding genome sequences of the ECC were subjected to pan-genome analysis, retrieving the core proteome followed by selecting potentially active vaccine candidates (outer membrane, periplasmic, and extracellular protein sequences) for epitope mapping. The vaccine were designed using pan-genome analysis and immunoinformatics (34). This type of vaccine is designed from the retrieved proteome and is more immunogenic and stable (35). Multi-epitope vaccines should consist of both B and T cell epitopes (36) thus the prediction of B cell epitopes (linear) and cytotoxic T lymphocytes is important (37). T lymphocytes contain T cell receptors (TCRs) that are responsible for the activation of immune response (38). T cell (B cell-derived) epitopes were predicted, and following examination of their immunogenic properties, those fulfilling the immunogenic criteria (antigenic, non-allergenic, non-toxic, soluble, and with adhesive property) were used to design a multi-epitope vaccine construct. The vaccine construct contains an epitope-epitope linker (GPGPG), an epitope-adjuvant linker (EAAAK), and an adjuvant (Cholera toxin B used in this study). Linkers play a vital role in the proper functioning of the vaccine by avoiding the overlapping of epitopes, aiding in stimulating the immune response, and conferring stability to the structure (39). Using the designed vaccine construct, a 3D model of the multi-epitope vaccine was predicted and subjected to loop modeling and refinement to predict the residues present in the loops of the structure. The quality of the model was analyzed by Ramachandran plot; 93.3% of the residues were in favored regions, and 0% of residues in the disallowed region, suggesting that the structure was of the highest quality (40). Disulfide engineering was performed in order to attain stability. Disulfide bonds were introduced into the structure at a region that was identified as instable, enhancing the thermostability of the structure. Thus, protein stability increased, which is very useful in biomedical and therapeutic industries. An increase in the stability of the protein used in therapeutics is of much importance as industrial enzymes with enhanced stability aid in better yield and have the potential to survive in unfavorable conditions compared to unmodified enzymes (41). Aggrescan, followed by CABS-flex analysis, was done to analyze the aggregation-prone regions in the multi-epitope vaccine. The peaks in the plot were stated to be aggregation-prone residues in the peptide-based vaccine model. Protein aggregation is involved in the boosting of immunogenicity. Aggregation-prone residues in a protein will affect its solubility, thus prediction of these regions is much valued to control protein deposition (42). CABS-flex, a coarse grind simulation, was performed to investigate and model the flexibility of the structure. The structure with the aggregation propensity was subjected to CABS-flex to inspect the structural flexibility. CABS-flex analysis can also be performed for the dock complexes to model and balance their property of flexibility (43).

## 5 Conclusion

In this article, computational base approaches were used for the identification of good vaccines to construct a multi-epitope vaccine against the *E. Cloacae* complex. In this study, highly immunogenic, non-toxic, human non-homologs, and non-allergic proteins were shortlisted for epitope mapping. To increase both humoral and cellular immunity, both B and T cell (MHC-I and MHC-II) epitopes were predicted. The epitopes predicted were checked for allergenicity, antigenicity, toxicity, and solubility. The filtered epitopes were linked together by GPGPG linkers and further joined to the adjuvant to make the vaccine construct more effective and potent. Disulfide engineering was done to maintain the structure’s stability. To induce a proper immune response, a vaccine construct should interact with the host immune cell receptors, thus the binding affinity of the vaccine with TLR-4 was also checked. Results reveal that the vaccine constructs have proper binding ability with the host immune cell receptors. The interaction stability of the vaccine and immune cells is crucial to generate long-lasting immunity, thus Molecular dynamics (MD) simulation analysis and binding free energies analysis were done for further validation of the docking results. Both the simulation and binding free energies estimation results revealed that there was proper binding stability of vaccine molecules with immune cell receptors. We observed from *in-silico* prediction that our designed vaccine construct can induce an immune response against the target pathogen, however, further experimental validation is strongly recommended.

## Data Availability

The original contributions presented in the study are included in the article/**Supplementary Material**. Further inquiries can be directed to the corresponding author.
