# Supplementary material for: Advanced vaccinomic, immunoinformatic, and molecular modeling strategies for designing Multi- epitope vaccines against the Enterobacter cloacae complex
Source: Front Immunol. 2024 Aug 16;15:1454394. doi: 10.3389/fimmu.2024.1454394 (PMC11362624; doi:10.3389/fimmu.2024.1454394)
Supplement: Supplementary file 1 [file DataSheet1.docx]

S1 **DNA sequence cloned in expression vector:**

ATGATCAAACTGAAATTCGGTGTTTTCTTCACCGTTCTGCTGTCTTCTGCTTACGCTCACGGTACCCCGCAGAACATCACCGACCTGTGCGCTGAATACCACAACACCCAGATCTACACCCTGAACGACAAAATCTTCTCTTACACCGAATCTCTGGCTGGTAAACGTGAAATGGCTATCATCACCTTCAAAAACGGTGCTATCTTCCAGGTTGAAGTTCCGGGTTCTCAGCACATCGACTCTCAGAAAAAAGCTATCGAACGTATGAAAGACACCCTGCGTATCGCTTACCTGACCGAAGCTAAAGTTGAAAAACTGTGCGTTTGGAACAACAAAACCCCGCACGCTATCGCTGCTATCTCTATGGCTAACGAAGCTGCTGCTAAAAACGCTAAACCGGAACCGGACGGTTCTGGTCCGGGTCCGGGTGGTGAAGACCGTGCTCTGGGTCAGACCGGTCCGGGTCCGGGTGACGACAACACCGCTTCTGCTCAGCACGGTCCGGGTCCGGGTCAGGCTGACGCTTGGGACATCAACCAGGGTCCGGGTCCGGGTAACACCAACAACGACAACTCTTCTTCTGGTCCGGGTCCGGGTCCGGCTCCGGCTCCGGCTCCGGAAGTTGGTCCGGGTCCGGGTGCTTACAACCAGAAACTGTCTGAAAAAGGTCCGGGTCCGGGTAACGCTGACACCCCGGGTTACCAGGCT

**Table S1: Protein-protein interaction between the Chain A, B, C, D (TLR-4) and Chain E (vaccine model).**

| Chains | No. of interface | | | Interface area (Å2) | | | No. of salt  bridges | No. of disulfide bonds | No. of hydrogen bonds | No. of non-bonded atoms |  |
| --- | --- | --- | --- | --- | --- | --- | --- | --- | --- | --- | --- |
|  |  |  |  |  |  |  |  |  |  |  |  |
|  |  |  |  |  |  |  |  |  |  |  |  |
| [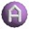](http://www.ebi.ac.uk/thornton-srv/databases/cgi-bin/pdbsum/GetPage.pl?pdbcode=b368&pdb_type=UPLOAD&code=090227&template=interfaces.html&o=RESIDUE&l=1)   \| [}{](http://www.ebi.ac.uk/thornton-srv/databases/cgi-bin/pdbsum/GetPage.pl?pdbcode=b368&pdb_type=UPLOAD&code=090227&template=interfaces.html&o=RESIDUE&l=1) \| \| --- \| | 8 | : | 8 | 516 | : | 508 | - | - | 2 | 20 |  |
| [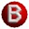](http://www.ebi.ac.uk/thornton-srv/databases/cgi-bin/pdbsum/GetPage.pl?pdbcode=b368&pdb_type=UPLOAD&code=090227&template=interfaces.html&o=RESIDUE&l=1)   \|  \| \| --- \| |  |  |  |  |  |  |  |  |  |  |  |
| [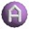](http://www.ebi.ac.uk/thornton-srv/databases/cgi-bin/pdbsum/GetPage.pl?pdbcode=b368&pdb_type=UPLOAD&code=090227&template=interfaces.html&o=RESIDUE&l=2)   \| [}{](http://www.ebi.ac.uk/thornton-srv/databases/cgi-bin/pdbsum/GetPage.pl?pdbcode=b368&pdb_type=UPLOAD&code=090227&template=interfaces.html&o=RESIDUE&l=2) \| \| --- \| | 26 | : | 19 | 903 | : | 1057 | 5 | - | 19 | 168 |  |
| [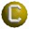](http://www.ebi.ac.uk/thornton-srv/databases/cgi-bin/pdbsum/GetPage.pl?pdbcode=b368&pdb_type=UPLOAD&code=090227&template=interfaces.html&o=RESIDUE&l=2)   \|  \| \| --- \| |  |  |  |  |  |  |  |  |  |  |  |
| [&](http://www.ebi.ac.uk/thornton-srv/databases/cgi-bin/pdbsum/GetPage.pl?pdbcode=b368&pdb_type=UPLOAD&code=090227&template=interfaces.html&o=RESIDUE&l=3) | 27 | : | 18 | 940 | : | 1084 | 6 | - | 24 | 162 |  |
| [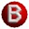](http://www.ebi.ac.uk/thornton-srv/databases/cgi-bin/pdbsum/GetPage.pl?pdbcode=b368&pdb_type=UPLOAD&code=090227&template=interfaces.html&o=RESIDUE&l=3)   \| [}{](http://www.ebi.ac.uk/thornton-srv/databases/cgi-bin/pdbsum/GetPage.pl?pdbcode=b368&pdb_type=UPLOAD&code=090227&template=interfaces.html&o=RESIDUE&l=3) \| \| --- \| |  |  |  |  |  |  |  |  |  |  |  |
| [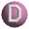](http://www.ebi.ac.uk/thornton-srv/databases/cgi-bin/pdbsum/GetPage.pl?pdbcode=b368&pdb_type=UPLOAD&code=090227&template=interfaces.html&o=RESIDUE&l=3)   \|  \| \| --- \| |  |  |  |  |  |  |  |  |  |  |  |
| [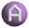](http://www.ebi.ac.uk/thornton-srv/databases/cgi-bin/pdbsum/GetPage.pl?pdbcode=b368&pdb_type=UPLOAD&code=090227&template=interfaces.html&o=RESIDUE&l=4)   \| [}{](http://www.ebi.ac.uk/thornton-srv/databases/cgi-bin/pdbsum/GetPage.pl?pdbcode=b368&pdb_type=UPLOAD&code=090227&template=interfaces.html&o=RESIDUE&l=4) \| \| --- \| | 15 | : | 10 | 504 | : | 580 | 2 | - | 4 | 64 |  |
| [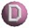](http://www.ebi.ac.uk/thornton-srv/databases/cgi-bin/pdbsum/GetPage.pl?pdbcode=b368&pdb_type=UPLOAD&code=090227&template=interfaces.html&o=RESIDUE&l=4)   \|  \| \| --- \| |  |  |  |  |  |  |  |  |  |  |  |
| [&](http://www.ebi.ac.uk/thornton-srv/databases/cgi-bin/pdbsum/GetPage.pl?pdbcode=b368&pdb_type=UPLOAD&code=090227&template=interfaces.html&o=RESIDUE&l=5) | 13 | : | 11 | 522 | : | 611 | 2 | - | 5 | 67 |  |
| [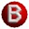](http://www.ebi.ac.uk/thornton-srv/databases/cgi-bin/pdbsum/GetPage.pl?pdbcode=b368&pdb_type=UPLOAD&code=090227&template=interfaces.html&o=RESIDUE&l=5)   \| [}{](http://www.ebi.ac.uk/thornton-srv/databases/cgi-bin/pdbsum/GetPage.pl?pdbcode=b368&pdb_type=UPLOAD&code=090227&template=interfaces.html&o=RESIDUE&l=5) \| \| --- \| |  |  |  |  |  |  |  |  |  |  |  |
| [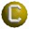](http://www.ebi.ac.uk/thornton-srv/databases/cgi-bin/pdbsum/GetPage.pl?pdbcode=b368&pdb_type=UPLOAD&code=090227&template=interfaces.html&o=RESIDUE&l=5)   \|  \| \| --- \| |  |  |  |  |  |  |  |  |  |  |  |
| [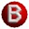](http://www.ebi.ac.uk/thornton-srv/databases/cgi-bin/pdbsum/GetPage.pl?pdbcode=b368&pdb_type=UPLOAD&code=090227&template=interfaces.html&o=RESIDUE&l=6)   \| [}{](http://www.ebi.ac.uk/thornton-srv/databases/cgi-bin/pdbsum/GetPage.pl?pdbcode=b368&pdb_type=UPLOAD&code=090227&template=interfaces.html&o=RESIDUE&l=6) \| \| --- \| | 18 | : | 16 | 718 | : | 770 | 7 | - | 15 | 144 |  |
| [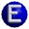](http://www.ebi.ac.uk/thornton-srv/databases/cgi-bin/pdbsum/GetPage.pl?pdbcode=b368&pdb_type=UPLOAD&code=090227&template=interfaces.html&o=RESIDUE&l=6)   \|  \| \| --- \| |  |  |  |  |  |  |  |  |  |  |  |
| [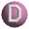](http://www.ebi.ac.uk/thornton-srv/databases/cgi-bin/pdbsum/GetPage.pl?pdbcode=b368&pdb_type=UPLOAD&code=090227&template=interfaces.html&o=RESIDUE&l=7)   \| [}{](http://www.ebi.ac.uk/thornton-srv/databases/cgi-bin/pdbsum/GetPage.pl?pdbcode=b368&pdb_type=UPLOAD&code=090227&template=interfaces.html&o=RESIDUE&l=7) \| \| --- \| | 15 | : | 11 | 638 | : | 591 | 1 | - | 8 | 98 |  |
| [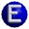](http://www.ebi.ac.uk/thornton-srv/databases/cgi-bin/pdbsum/GetPage.pl?pdbcode=b368&pdb_type=UPLOAD&code=090227&template=interfaces.html&o=RESIDUE&l=7)   \|  \| \| --- \| |  |  |  |  |  |  |  |  |  |  |  |

**Table S2: Energies calculated for Dock-complex (vaccine-MHCI). Energies at the center of the cluster and the complex with lowest energy in the cluster listed in column named weighted score.**

| **Cluster** | **Members** | **Representative** | **Weighted Score** |
| --- | --- | --- | --- |
| **0** | 91 | Center | -849.8 |
|  |  | Lowest Energy | -886.7 |
| **1** | 89 | Center | -732.7 |
|  |  | Lowest Energy | -783 |
| **2** | 60 | Center | -677.7 |
|  |  | Lowest Energy | -760.9 |
| **3** | 43 | Center | -823.8 |
|  |  | Lowest Energy | -869.4 |
| **4** | 43 | Center | -700.7 |
|  |  | Lowest Energy | -772.9 |
| **5** | 41 | Center | -668.1 |
|  |  | Lowest Energy | -764.9 |
| **6** | 37 | Center | -703.3 |
|  |  | Lowest Energy | -951 |
| **7** | 26 | Center | -756.1 |
|  |  | Lowest Energy | -756.1 |
| **8** | 24 | Center | -792.2 |
|  |  | Lowest Energy | -792.2 |
| **9** | 23 | Center | -860.8 |
|  |  | Lowest Energy | -860.8 |

**Table S3: Energies calculated for Dock-complex (vaccine-MHCII). Energies at the center of the cluster and the complex with lowest energy in the cluster listed in column named weighted score.**

| **Cluster** | **Members** | **Representative** | **Weighted Score** |
| --- | --- | --- | --- |
| **0** | 125 | Center | -900.1 |
|  |  | Lowest Energy | -995.6 |
| **1** | 119 | Center | -803.2 |
|  |  | Lowest Energy | -898.3 |
| **2** | 97 | Center | -805.9 |
|  |  | Lowest Energy | -946.8 |
| **3** | 77 | Center | -836.6 |
|  |  | Lowest Energy | -964.2 |
| **4** | 68 | Center | -804.3 |
|  |  | Lowest Energy | -921.4 |
| **5** | 60 | Center | -902.7 |
|  |  | Lowest Energy | -1082.7 |
| **6** | 60 | Center | -875.6 |
|  |  | Lowest Energy | -1027.1 |
| **7** | 55 | Center | -941.2 |
|  |  | Lowest Energy | -1048.4 |
| **8** | 41 | Center | -970.4 |
|  |  | Lowest Energy | -1020.8 |
| **9** | 38 | Center | -1026.2 |
|  |  | Lowest Energy | -1102.4 |

**Table S4: Energies calculated for Dock-complex (vaccine-TLR-4). Energies at the center of the cluster and the complex with lowest energy in the cluster listed in column named weighted score.**

| **Cluster** | **Members** | **Representative** | **Weighted Score** |
| --- | --- | --- | --- |
| **0** | 82 | Center | -798.5 |
|  |  | Lowest Energy | -883.6 |
| **1** | 49 | Center | -738.7 |
|  |  | Lowest Energy | -738.7 |
| **2** | 40 | Center | -811.7 |
|  |  | Lowest Energy | -811.7 |
| **3** | 40 | Center | -665.4 |
|  |  | Lowest Energy | -754.2 |
| **4** | 37 | Center | -853.4 |
|  |  | Lowest Energy | -853.4 |
| **5** | 37 | Center | -823.3 |
|  |  | Lowest Energy | -823.3 |
| **6** | 33 | Center | -678.8 |
|  |  | Lowest Energy | -771.5 |
| **7** | 31 | Center | -703.1 |
|  |  | Lowest Energy | -803 |
| **8** | 23 | Center | -707.5 |
|  |  | Lowest Energy | -743.6 |
| **9** | 23 | Center | -706.5 |
|  |  | Lowest Energy | -729.1 |
